# Sulfur - fluorine bond in PET radiochemistry

**DOI:** 10.1186/s41181-017-0028-6

**Published:** 2017-07-17

**Authors:** Giancarlo Pascali, Lidia Matesic, Bo Zhang, Andrew T. King, Andrea J. Robinson, Alison T. Ung, Benjamin H. Fraser

**Affiliations:** 1 Australian Nuclear Science and Technology Organisation, New South Wales, Australia; 2 Brain and Mind Centre, University of Sydney, New South Wales, Australia; 3 School of Chemistry, Monash University, Victoria, Australia; 4 School of Mathematical and Physical Sciences, University of Technology Sydney, Sydney, NSW Australia

**Keywords:** Sulfur-fluorine bond, Sulfonyl fluoride, ^18^F, PET, Fluoride relay, Fluorosulfate, Deoxyfluorination, Sulfurhexafluoride

## Abstract

The importance of the sulfur-fluorine bond is starting to increase in modern medicinal chemistry literature. This is due to a better understanding of the stability and reactivity of this moiety depending on the various oxidation states of sulfur. Furthermore, several commercial reagents used for mild and selective fluorination of organic molecules are based on the known reactivity of S-F groups. In this review, we will show how these examples are translating into the ^18^F field, both for use as stable tags in finished radiopharmaceuticals and as mildly reactive fluoride-relay intermediates. Finally, we also discuss current opportunities where examples of non-radioactive S-F applications/chemistry may be translated into future ^18^F radiochemistry applications.

## Background

The field of ^18^F radiochemistry is constantly investigating the utilization of novel fluorinated pharmaceuticals, and the radiofluorination processes that can lead to their production. The sulfur-fluorine bond is starting to gain importance, both for its presence in the final structures and for utilisation as a “fluoride relay” species. This perspective review will show how some of these applications are currently utilized and reported in ^18^F literature, and it will also provide examples from non-radioactive fluorine chemistry that could inspire future radiofluorine applications.

### Features, reactivity and uses of the S-F bond

**Fig. 1:**
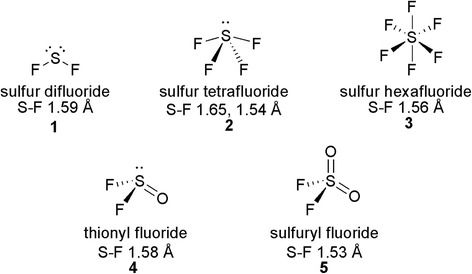
S-F bond lengths in different model molecules

The physiochemical characteristics of the S-F bond are highly dependent upon the sulfur atom oxidation state and the nature of the substituents attached to the central sulfur atom. The sulfur fluoride series of sulfur difluoride (SF_2_), sulfur tetrafluoride (SF_4_) and sulfur hexafluoride (SF_6_) are instructive in demonstrating these trends (Fig. 1). The S-F bond lengths for SF_2_ (1.59 Å), SF_4_ (1.65 Å, 1.54 Å) and SF_6_ (1.56 Å) vary according to shortening effects from decreasing electron density on sulfur and lengthening effects due to increasing steric encumbrance (Cotton and Wilkinson G. 1999). Even more strikingly, their respective S-F bond reactivity and lability vary quite significantly. SF_2_ (**1**, oxidation state II) is highly unstable, must be synthesised at low pressure, and decomposes rapidly to 1,1,1, 2-tetrafluorodisulfane (FSSF_3_). SF_4_ (**2**, oxidation state IV) is a stable gas at room temperature and pressure, but is extremely reactive and hydrolyses instantly upon exposure to moisture, liberating hydrogen fluoride (HF) and sulfur dioxide. SF_4_ is utilized in non-radioactive fluorination chemistry to convert carbonyl (C=O) and phosphoryl (*P* = O) groups to CF_2_ and PF_2_ groups respectively. Finally, sulfur hexafluoride (**3**, oxidation state VI) is highly stable and inert under all but the most extreme reaction conditions. This stability is exploited in its application as an electrical insulating material, a gas for detached retina surgery on humans and as an air-flow tracer. The introduction of oxygen (S = O groups) produces similar effects to the successive introduction of fluorine. Thionyl fluoride (**4**, SOF_2_) and sulfuryl fluoride (**5**, SO_2_F_2_) again show similar trends in bond length to the sulfur fluoride series. The S-F bond in SOF_2_ (1.58 Å) is longer than in SO_2_F_2_ (1.53 Å). SOF_2_ (oxidation state IV), however, reacts rapidly with moisture to liberate SO_2_ and HF and is not used extensively in fluorination chemistry. SO_2_F_2_ (oxidation state VI) by contrast is relatively stable to hydrolysis (up to temperatures of 150 °C) and finds application as a fumigant and insecticide. Focusing on the sulfur (VI) oxidation state, a comparison of sulfuryl fluoride (SO_2_F_2_) with the corresponding chloride (SO_2_Cl_2_) shows the S-F bond is significantly stronger (~40 kcalmol^-1^) (Kiang and Zare RN 1980) than the S-Cl bond, more resistant to hydrolysis and thermolysis (Kice and Lunney 1975), unreactive towards reductive elimination (unlike the S-Cl bond) (Chatgilialoglu C. Sulfinyl Radicals. In: Sulphones and Sulphoxides 1988) and requires very specific conditions to facilitate nucleophilic substitution. Given these unique characteristics, functional groups of this class have attracted considerable interest from researchers and in this review we summarise the current literature describing [^18^F] sulfur-fluorine radiochemistry.

The current literature can be divided into two modes of utilization. In the first approach, the S-^18^F moiety is present in the final target molecule and procedures have been developed in order to create this bond in a selective and mild fashion. In the second case, the reactive feature of the S-F bond is exploited in order to relay, in a specific fashion, the ^18^F label to an appropriate precursor. In this approach, the S-^18^F bond is created in an intermediate molecule and not present in the final target compound. Given the physicochemical trends discussed previously, most examples discussed herein belong to the sulfur (VI) oxidation family. It is almost certain, but perhaps we may be rebutted, that sulfur (II) fluoride containing functional groups are far too reactive to ever find application in fluorine-18 radiochemistry. This is not the case, however, for sulfur (IV) fluoride containing functional groups, which have many known applications in regular fluorination chemistry, and may begin to be utilized as transfer agents in fluorine-18 radiochemistry.

### Therapeutics and biological tools incorporating sulfonyl fluoride moieties

**Fig. 2:**
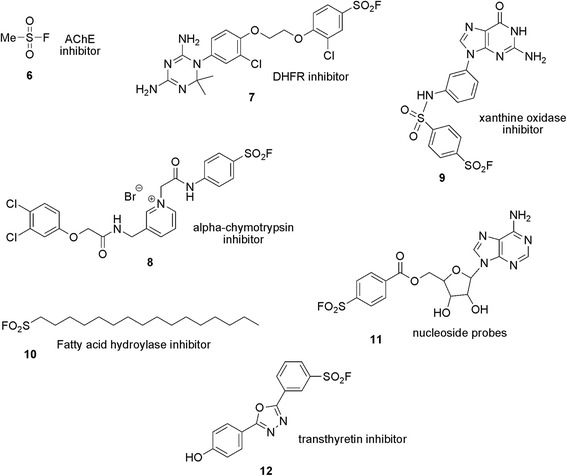
Examples of molecules containing sulfonyl fluoride moieties

Radiochemists and nuclear medicine clinicians unfamiliar with sulfur fluoride chemistry may, with some reason, anticipate that this type of functional group would be too reactive for therapeutic or in vitro tool applications. This is not surprising as no [^18^F]sulfonyl fluoride labelled compounds have been approved for use in humans or have been evaluated in clinical trials as PET diagnostics. Non-radioactive [^19^F] sulfonyl fluorides, however, owing to their unique stability and relatively mild reactivity, have found several applications as pharmacological therapeutics and in vitro tools (Moss DE. Sulfonyl fluorides for the treatment of alzheimer’s disease 1996; Baker et al. 1970; Baker and Vermeulen 1970; Baker and Hurlbut 1969; Baker and Wood 1969; Karanian D a et al. 2005; Colman 1997; Grimster et al. 2013). The application depends upon the ability of the sulfonyl fluoride functional group to remain inert when attached to a biological vector and circulating in a biological fluid (in vitro or in vivo). Once the vector finds the target, the sulfonyl fluoride comes to life and reacts irreversibly and covalently with the target. This phenomenon is referred to as affinity-driven activation (Moss DE. Sulfonyl fluorides for the treatment of Alzheimer’s disease 1996; Baker et al. 1970; Baker and Vermeulen 1970; Baker and Hurlbut 1969; Baker and Wood 1969; Karanian D a et al. 2005; Colman 1997; Grimster et al. 2013). Examples of this class of compound include methane sulfonyl fluoride (MSF, **6**, Fig. 2) – a potent inhibitor of acetylcholinesterase (AChE) – which has been evaluated in phase I and phase II clinical trials for the treatment of Alzheimer’s disease. MSF is effective in reducing Alzheimer’s symptoms and a patent for the treatment of dementia has been granted (Moss DE. Sulfonyl fluorides for the treatment of alzheimer’s disease 1996). Looking back further into the history of sulfonyl fluorides, the group of Baker developed several series of compounds that were evaluated as covalent inhibitors for multiple disease targets. These included sulfonyl fluoride **7** which was based on the structure of the dihydrofolate reductase (DHFR) inhibitor (NSC 139105) (Baker et al. 1970; Baker and Vermeulen 1970). Irreversible, selective inhibitors of DHFR have potential as anti-bacterial and anti-cancer therapeutics. The same group have also published irreversible, covalent inhibitors of α-chymotrypsin based upon the sulfonyl fluoride **8** (Baker and Hurlbut 1969). A series of candidates including sulfonyl fluoride **9** were also evaluated as potential irreversible inhibitors of xanthine oxidase but were only found to cause reversible inhibition. Xanthine oxidase inhibitors are used for the treatment of hyperuricemia and related medical conditions including gout (Baker & Wood 1969). More recently the group of Bahr evaluated palmitylsulfonyl fluoride **10** as an inhibitor of fatty acid amide hydrolase (FAAH) which can help defend against several disease states including excitotoxic brain damage (Karanian D a et al. 2005). In terms of in vitro tools, nucleoside probes such as sulfonyl fluoride **11** have been published by the group of Colman as agents for selective labelling of NAD and ATP binding sites in numerous proteins (Colman 1997). More recently the groups of Kelly and Sharpless have shown that sulfonyl fluoride functionalised 1, 3, 4-oxadiazoles **12** irreversibly bind to the active site of transthyretin and prevent misfolding of the protein. This work could have significant impact for the treatment of amyloid diseases including Alzheimer’s and Parkinson’s disease (Grimster et al. 2013).

## Sulfur-[^18^F] fluorine radiolabelled reagents and compounds

### [^18^F]Sulfonyl fluorides

**Fig. 3:**
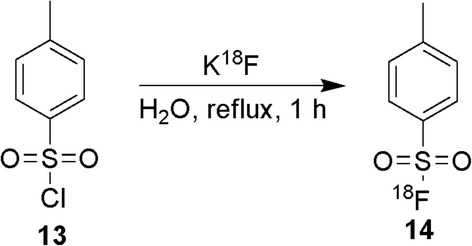
Radiosynthesis of [^18^F] tosyl fluoride from tosyl chloride

**Fig. 4:**
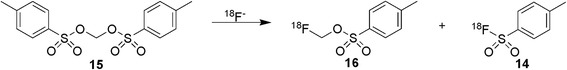
Radiosynthesis of [^18^F] tosyl fluoride as a by-product of the reaction between *bis* (tosyloxy) methane and [^18^F] fluoride

The first account of the sulfur-[^18^F] fluorine bond was reported in the 1970s by de Kleijn and co-workers (De Kleijn et al. 1975; De Kleijn and van Zantan 1977). The preparation of [^18^F] tosyl fluoride (**14**) was reported by reacting tosyl chloride with the potassium [^18^F] fluoride in water, in an unreported yield (Fig. 3). The radiosynthesis of [^18^F] tosyl fluoride was also reported by Neal et al. (Neal et al. 2005); however, in this case, the compound was an undesired by-product from the reaction of *bis* (tosyloxy) methane (**15**) with [^18^F] fluoride to form [^18^F] fluoromethyltosylate (**16**, Fig. 4). The formation of the [^18^F] tosyl fluoride by-product was highly variable in the reaction. The ratio of [^18^F] fluoromethyltosylate : [^18^F] tosyl fluoride changed from 68:32 to 9:91 when the amount of Kryptofix 222 in the radiochemical reaction was reduced from 18 mg to 1.5 mg. The use of tetrabutylammonium bicarbonate as an activating agent also led to high yields of [^18^F] tosyl fluoride being formed as the by-product (85%). The authors were also able to increase the yield of the desired [^18^F] fluoromethyltosylate by adding water (up to 10% v/v) to the reaction, which was claimed to hydrolyze the [^18^F] tosyl fluoride. This justification is surprising, as sulfonyl fluorides are known to be synthesized from the chlorides even under aqueous conditions (Davies and Dick 1931). A more plausible explanation may rely on hardness-softness of the site of nucleophilic attack, but no data are currently available to support such hypothesis.

**Fig. 5:**
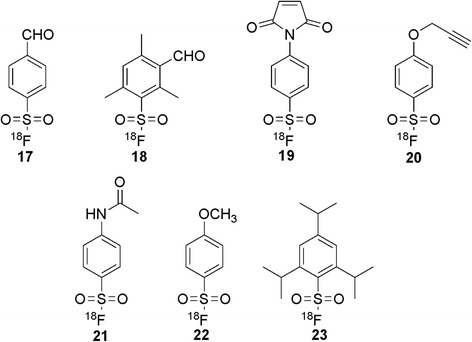
Selected aryl [^18^F]sulfonyl fluorides

In recent years, attention has turned to using [^18^F]sulfonyl fluorides as prosthetic groups for ligands which can be used for PET imaging. In 2012, Inkster et al. reported four [^18^F]sulfonyl fluorides containing 4-formyl-, 3-formyl-, 4-maleimido- and 4-oxyalkynyl moieties (**17–20**, Fig. 5), synthesised in a 1:1 mixture of an organic solvent and an aqueous solution of caesium carbonate (Inkster et al. 2012). The 3-formyl analogue (**18**) was selected, due to its favourable stability in PBS buffer over 2 h, to be conjugated to the nonapeptide bombesin. The resulting radiolabelled peptide was stable in 10% DMSO in PBS buffer over 2 h at physiological temperature and pH, however, when analyzed in mouse serum, this product was found to only be 55% intact after 15 min, indicating defluorination was occurring.

Shortly after, Matesic et al. investigated the suitability of twelve aryl [^18^F] sulfonyl fluorides for applications in ^18^F-radiochemistry (Matesic et al. 2013). The [^18^F] sulfonyl fluorides were prepared by reacting the corresponding sulfonyl chlorides with [^18^F] fluoride under microfluidic conditions. These molecules bore neutral, electron-donating and electron-withdrawing functional groups. Additionally, sulfonyl chlorides containing varying degrees of steric bulk and a heterocyclic sulfonyl chloride were evaluated. Under microfluidic synthesis conditions, the [^18^F] sulfonyl fluorides could be prepared in less than 60 s at temperatures between 30–180 °C using a 0.5 mg/mL solution of the sulfonyl chloride precursor. The exception to this rule was the electron-withdrawing [^18^F] 4-nitrobenzenesulfonyl fluoride, which could not be produced at all using the parameters above. Interestingly, in the presence of 5% water in the reaction mixture, the compound could be formed in 91% radiochemical yield (RCY) at 30 °C (Pascali et al. 2014). This result is not unprecedented as several reports in recent years have described increased RCY when the molecules are prepared in solutions containing varying percentages of water (Sergeev et al. 2015). The reaction parameters of all analogues could be fine-tuned to produce >75% RCY at 30–180 °C by increasing precursor concentration, adding water, altering residence time, etc. The compounds were monitored for stability in buffers, before the stability of the leading candidates (**21–23**, Fig. 5) were evaluated in rat serum. After 2 h at physiological temperature in serum, the [^18^F] 2, 4, 6-triisopropylbenzenesulfonyl fluoride (**23**) was still 95% intact, suggesting that steric bulk around the sulfur-[^18^F] fluorine bond was more important in protecting the bond from hydrolysis, than the electron density of the molecule.

**Fig. 6:**
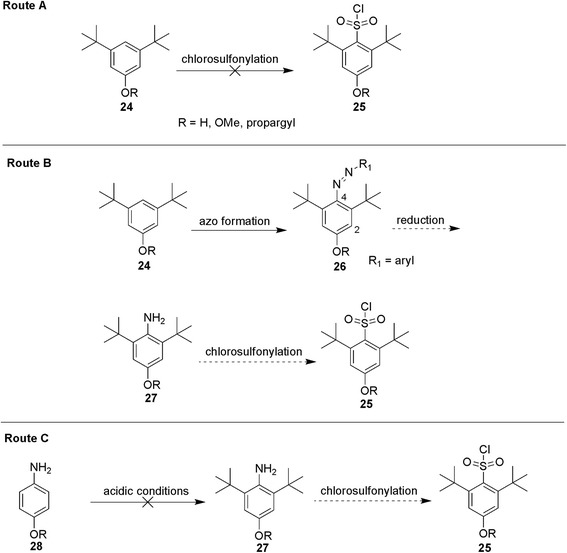
Possible synthetic routes towards the formation of a sterically hindered sulfonyl chloride

Following these encouraging preliminary results, it was envisaged that a sterically hindered sulfonyl chloride could be produced as a synthon to be subsequently radiolabelled with [^18^F] fluoride and conjugated to a peptide or protein. Ideally, the radiosynthon would contain an alkynyl pendant, allowing it to undergo bioconjugation with an azido modified macromolecule using click chemistry (Roberts et al. 2015). The radiosynthon would contain di-*tert* butyl groups to provide additional protection against hydrolysis of the sulfur-[^18^F] fluorine bond and could be formed using a variety of routes (Fig. 6). In our initial testing (unpublished data) of the synthesis of appropriate precursors (King AT 2016), we subjected the di-*tert* butyl analogue **24** (Route A, Fig. 6) to acidic conditions using chlorosulfonic acid in an attempt to produce the desired sulfonyl chloride **25**, however the reaction was unsuccessful. Alternatively, **24** could be used to synthesize an azo intermediate (**26**, Route B, Fig. 6), that could be reduced to the amino derivative **27** and converted to a sulfonyl chloride using a diazonium ion intermediate. Intriguingly, the azo formation did not proceed in the 4-position of the molecule, but rather in the 2-position, as confirmed by 2D NMR experiments. It is postulated that the azo group preferentially attached to the 2-position of the aromatic ring due to the decreased amount of steric hindrance compared to the 4-position. The third route (Route C, Fig. 6) involved a Friedel-Crafts alkylation on an amine (**28**), followed by chlorosulfonylation through a diazonium intermediate. A recent literature example reported the Friedel-Crafts alkylation of 4-methoxyaniline and diphenylmethanol to introduce two diphenyl groups around the amine bond (Meiries et al. 2013). Applying this strategy to introduce two *tert*-butyl groups in **27** was unsuccessful. The most likely explanation was the absence of the carbocation generation required for electrophilic substitution. Compounds containing amino substituents are also sometimes known to be *meta*-directing due to the acidic reaction conditions converting the amine into an ammonium ion (McMurry J 2012). However, in this case no addition of the *tert*-butyl groups was observed, as determined by NMR and mass spectrometry. These three synthetic routes highlight the complexities in synthesizing a sterically hindered sulfonyl chloride – the addition of the sulfonyl chloride moiety in between two sterically bulky functional groups (Routes A and B, Fig. 6) can be just as difficult as introducing sterically bulky groups onto a precursor molecule (Route C, Fig. 6). These results indicate that the design and synthesis of a sterically bulky sulfonyl chloride synthon require further investigation in the future.

Potentially inspired by the feasibility of using microfluidic devices to radiolabel small molecules with ^18^F, Fiel et al., revisited the radiosynthesis of the [^18^F] 3-formylbenzenesulfonyl fluoride **18** in Fig. 5, using a magnetic droplet microfluidic (MDM) platform (Fiel et al. 2015). The platform consists of a Teflon sheet mounted on a stage, with a robotic arm beneath the Teflon sheet which controls the movement of the magnetic particles on the stage. [^18^F] Fluoride preconcentration and subsequent radiolabelling were performed on the same platform, in contrast to most traditional radiolabelling procedures whereby the [^18^F] fluoride is preconcentrated in a vial external to the microfluidic device. The authors were able to radiolabel the 3-formyl analogue **18** in *tert*-butanol in 5 min at room temperature using a total volume of 100 μL. The RCY (72 ± 1%, *n* =3) was comparable with that reported by Inkster et al. (73 ± 7%) (Inkster et al. 2012), however, the reaction time had decreased from 15 min to 5 min.

**Fig. 7:**
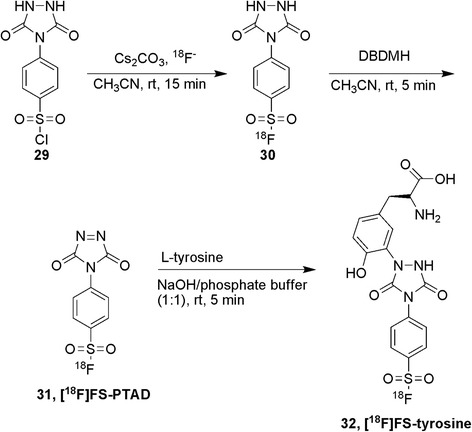
Radiosynthesis of the [^18^F] FS-PTAD radiosynthon and its conjugation to tyrosine

Another sulfonyl fluoride bearing radiosynthon has been reported by Al-Momani et al. (Al-Momani et al. 2015). The radiosynthon, [^18^F] FS-PTAD (**31**) could be synthesised in 91% RCY by converting the sulfonyl chloride moiety on urazole **29** to the corresponding sulfonyl [^18^F] fluoride (**30**), followed by oxidation using 1, 3-dibromo-5,5-dimethylhydantoin (DBDMH) (Fig. 7). [^18^F] FS-PTAD was conjugated to model substrates in moderate to good RCY under mild aqueous conditions (phosphate buffer, pH 7), however, basic conditions (sodium hydroxide, pH 9–10) were required to conjugate [^18^F] FS-PTAD to tyrosine. The [^18^F] FS-tyrosine (**32**) was >95% intact after 2 h in a 8% ethanol/PBS buffer solution, without the requirement of steric bulk around the sulfur-[^18^F] fluorine bond to prevent it from hydrolysis, as suggested previously (Matesic et al. 2013). The authors also demonstrated moderate uptake of [^18^F] FS-tyrosine into two human glioblastoma and one rat glioma cell lines, which warrants further exploration.

### [^18^F] Ethenesulfonyl fluoride (ESF)

**Fig. 8:**
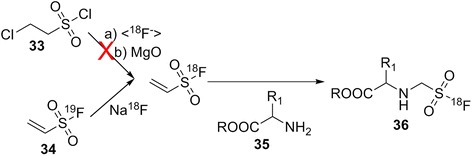
Scheme for c.a. [^18^F] ESF synthesis and use for radioconjugation to model amino acids

Still in the field of sulfonyl fluorides, ethenesulfonyl fluoride (ESF, **34**) has been reported as one of the strongest Michael acceptors (Chen et al. 2016) and it has been reported to react with various “soft” nucleophiles under mild reaction conditions (Krutak et al. 1979). Consequently, [^18^F] ESF has significant potential to be used as a prosthetic group to radiolabel targets that contain lysine, cysteine residues or other ‘soft’ nucleophilic groups. The small size and hydrophilicity of [^18^F] ESF makes it ideal for radiolabelling proteins and polypeptides without drastically altering its pharmacological and polarity features, especially in comparison with currently used larger hydrophobic prosthetic groups. Our group has recently reported (Zhang et al. 2016) the first synthesis of radioactive [^18^F] ESF using a carried added (c.a.) process. We discovered that the route reported for the synthesis of non-radioactive ESF, employing fluorination and dehydrochlorination of 2-chloroethanesulfonyl chloride (**33**), was unsuccessful with [^18^F] fluoride. Therefore, a simple ^19^F/^18^F substitution reaction was attempted, and >80% RCY of [^18^F] ESF was obtained in diluted saline at 130 °C, employing a simple alumina cartridge purification approach (Fig. 8). This prosthetic group was employed in reaction with DMF solutions of seven model carboxy-protected amino acids (**35**), and the corresponding radioconjugate (**36**) was obtained in typically >40% yield at room temperature. Current investigations are undergoing towards synthesis of no carrier added (n.c.a.) [^18^F] ESF, its utilization for radioconjugation of proteins and polypeptides and the in vivo stability of the obtained products.

### [^18^F] Fluorosulfate ion

**Fig. 9:**
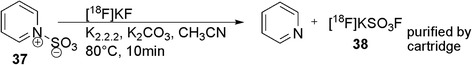
Synthesis of potassium [^18^F] fluorosulfate

Recent work into the utilization of [^18^F] fluorosulfate ion (**38**) as a PET imaging agent for the Na/I symporter (NIS) has been published by the group of Gee (Khoshnevisan et al. 2017). The function of NIS, a small anion transporter system, is important in the accumulation of iodide in thyroid follicles for the synthesis of hormones. The imaging of this system paves the way for the understanding of hormonal-related disease, as well as for assessing the efficacy of thyroid-targeted radiotherapies. Several radioactive anions have been used so far, mainly radioactive iodide ([^131/123/124^I] I^-^) or [^99m^Tc] TcO_4_^-^, with [^186/188^Re] ReO_4_^-^ and [^18^F] BF_4_^-^ evaluated more recently (Ahn 2012; Dadachova et al. 2002; Weeks et al. 2011). The use of [^18^F] BF_4_^-^ has demonstrated superior imaging features and optimal half-life characteristics, but the synthesis route afforded the radiotracer in low molar activity (1-8.8 MBq/nmol (Jiang et al. 2016)). Subsequently, the same group investigated different anions and their potential labelling with ^18^F. They have identified that at least three other fluorinated anions could be used for NIS imaging, due to their inhibitory potency: SO_3_F^-^, PO_2_F_2_^-^ and PF_6_^-^ (this last example being the most potent). On the basis of availability of amenable and practical radiosynthesis routes, [^18^F] SO_3_F^-^ (**38**) was selected as a suitable candidate for novel NIS imaging. Compound **38** was synthesized by reaction of K_222_/[^18^F] KF dried complex with SO_3_-pyridine adduct (**37**, Fig. 9) in CH_3_CN. The radiochemical yield was up to 65% in 10 min at 80 °C. The purification was simply performed by passing the water diluted reaction mixture through neutral Al and QMA cartridges. [^18^F] SO_3_F^-^ was recovered by eluting the QMA cartridge with 0.9% NaCl isotonic solution, providing the radiotracer in 31% RCY, >95% RCP, >48 MBq/nmol molar radioactivity and overall radiosynthesis time of <60 min. Radiotracer identity was confirmed by ion chromatography and residual amounts of pyridine, K_222_ and SO_4_^2-^ were also determined.

Tracer stability studies confirmed a RCP of >95% (after 4 h) in multiple media including formulation media, acidic environment and serum. Specific uptake of the tracer was confirmed in vitro in NIS-expressing cell lines, as well as in vivo in healthy mice using PET-CT. During the imaging studies, a steady increase of radioactivity was noticed in bones, especially after 30 min; however, the authors believe that this metabolism will likely not hamper the utility of [^18^F] SO_3_F^-^ for NIS imaging. This work represented the first ever PET-CT imaging of a compound bearing an S-^18^F bond.

### [^18^F]deoxyfluorinating agents

**Fig. 10:**
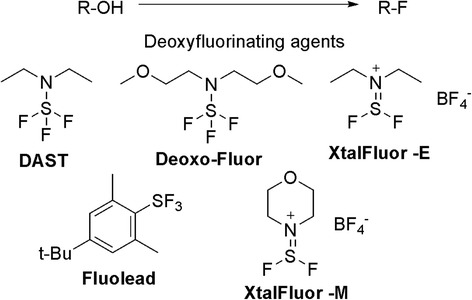
Deoxyfluorinating reagents containing S-F moieties

Despite the abundance of non-radioactive deoxyfluorination reactions published to date only a very limited number involving ^18^F have been reported up to date. From the chemical literature, there are multiple examples of deoxyfluorinating reagents containing an S-F bond (Fig. 10), such as DAST (diethylaminosulfurtrifluoride), Deoxo-Fluor (2-methoxy-*N*-(2-methoxyethyl)-*N*-(trifluoro-λ4-sulfanyl)ethanamine), XtalFluor –E and –M ((difluoro-λ4-sulfanylidene)(diethyl)ammonium tetrafluoroborate and 4-(difluoro-λ4-sulfanylidene)morpholin-4-ium tetrafluoroborate) and Fluolead (4-*tert*-butyl-2,6-dimethylphenylsulfur trifluoride) (Ni et al. 2014). The structure of these molecules features more than one fluorine atom, therefore posing limitations and posing significant challenges in regard to their radiosynthesis and the obtainable molar radioactivity.

**Fig. 11:**
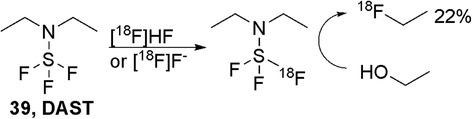
Synthesis and use of [^18^F] DAST

Nonetheless, [^18^F] DAST was synthesized and used (Straatmann and Welch 1977) in the fluorination of short chain model alcohols. In this 1977 paper, the group of Welch tested three routes for the synthesis of this fluorinating reagent: (a) a *de-novo* synthesis of [^18^F] DAST from target produced [^18^F] SF_4_, (b) ^18^F/^19^F isotopic exchange on DAST with [^18^F] F_2_ and finally, (c) an exchange via treatment with [^18^F] HF. The best route was determined to be (c), that yielded [^18^F] DAST (**39**) in >80% RCY and was then used to deoxyfluorinate methanol, ethanol and ethylene glycol, with RCY of 20%, 25% and 12% respectively (Fig. 11). Given that the radiofluorinating reagent produced bears 3 fluorine atoms and therefore can provide a maximum RCY of 33%, the reported yields for the employed [^18^F] fluoroalkanes are relatively high. The authors however recognize that the achievable molar radioactivity is particularly low, due to the use of isotopic exchange reactions.

More recent literature has indicated that sulfonyl fluorides are viable as [^18^F] deoxyfluorinating reagents (Takamatsu et al. 2002; Guo and Ding 2015), and indeed some applicative value has been demonstrated by perfluorobutanesulfonyl fluoride (PFBS) (Vorbrüggen H. The conversion of primary or secondary alcohols with nonaflyl fluoride into their corresponding inverted fluorides. Synthesis (Stuttg) 2008; Bennua-Skalmowski and Vorbruggen 1995). Inspired by these contributions, Nielsen et al. have reported the synthesis and use of [^18^F] PyFluor (2-pyridinesulfonyl fluoride, **41**) (Nielsen et al. 2015). In this work, the authors first studied a range of sulfonyl fluorides (comprising PFBS) to fluorinate a model alcohol, using 1.1 eq of fluorinating reagent and 2 eq of 1,8-diazabicyclo-[5.4.0] undec-7-ene (DBU) or 7-methyl-1,5,7-triazabicyclo [4.4.0] dec-5-ene (MTBD) as a base. This preliminary set of experiments revealed PyFluor as the most promising reagent, with the highest fluorination yield and selectivity (towards dexoyfluorination reaction). The postulated mechanism involves the formation of an intermediate sulfonate between Pyfluor and the alcohol, and its subsequent fluorination by nucleophilic substitution by the liberated fluoride ion. DBU or MTBD act as Brønsted bases and are proposed to help stabilize the developing fluoride ion, rendering it available to nucleophilic reaction. The wide scope demonstrated, allowed for Pyfluor to now be commercially available through Sigma-Aldrich as a deoxyfluorinating reagent.

**Fig. 12:**
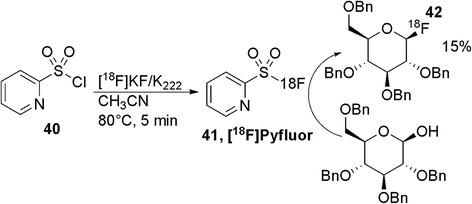
Synthesis and use of [^18^F] Pyfluor

In the same contribution, [^18^F] Pyfluor (**41**) was synthesized from the respective sulfonyl chloride (**40**) in CH_3_CN at 80 °C, giving the radio-synthon in 88% RCY in 5 min (Fig. 12). The addition of a benzyl protected tetrahydro-2*H*-pyran-pyran-2-ol to the same pot gave a 15% RCY of the desired deoxyradiofluorinated compound (**42**) to be obtained in 20 min. Currently, this has been the only reported radiofluorine application of the Pyfluor approach. Even if promising, a wider utilization of this approach might be hindered by the difficult access to the needed labelling precursor **40**.

### [^18^F] SF_6_ production and uses

[^18^F] Fluoride atoms produced by the irradiation of SF_6_ gas through the^19^F(n,2n)^18^F nuclear reaction in fast neutron generators were first reported in the 1970s (Colebourne and Wolfgang 1963). Even though these ^18^F atoms lost the majority of their kinetic energy in the presence of SF_6_ (Smail et al. 1972), they were still able to undergo addition onto olefins, ethylene and acetylene to produce the ^18^F-fluorinated analogues (Williams and Rowland 1971), and this observation was even proposed as a useful method for scavenging undesired ^18^F atoms. The production of [^18^F] SF_6_ remained largely unexplored in the radiochemistry literature for 40 years, until Gómez-Vallejo reported the cyclotron production of [^18^F] SF_6_ in 2016 (Gómez-Vallejo et al. 2016). In this method, the authors verified that new cyclotrons are able to exploit the (p, pn) route to obtain c.a. fluorinated gases (in this test CF_4_ was also produced), but the yield obtained are insufficient for imaging uses. Therefore, they optimized a double irradiation method, in which [^18^O] O_2_ was used as target material in the first irradiation, and CF_4_ or SF_6_ were used to fill the target in a second irradiation. In this way, almost 7 GBq of [^18^F] SF_6_ was produced using an integrated current of 4 μA and the authors hinted at the possibility of using such ^18^F-radiolabelled fluorinated gases for in vivo PET imaging assessment of lung ventilation. The availability of [^18^F] SF_6_ may also turn useful in multimodal imaging approaches, due the current use of SonoVue® (from Bracco), an SF_6_ microbubble formulation, as an ultrasound contrast imaging agent.

## Future opportunities

### Desulfurization of aromatic sulfonyl fluorides

**Fig. 13:**
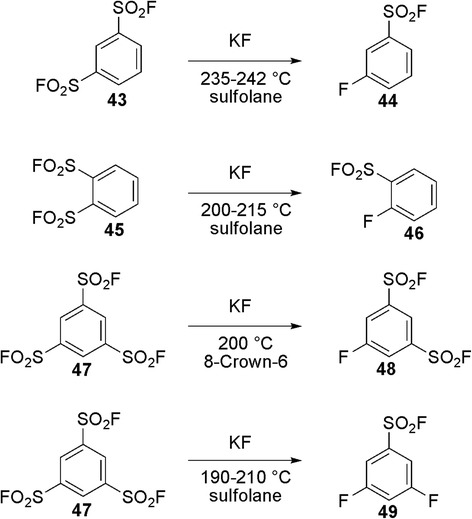
Synthesis of arylfluorides by desulfurization of sulfonylfluorides

The first efficient synthesis of an aryl fluoride via desulfurization of the corresponding aromatic sulfonyl fluoride was reported by Van Der Puy (Van der Puy 1988). Although this transformation was well known for sulfonyl chloride and sulfonyl bromide substrates (Miller and Walling 1957; Blum and Scharf 1970) it had never been optimized for sulfonyl fluorides. In this work (Fig. 13) various benzene-1,3-disulfonyl fluorides (**43**) and benzene-1,2-disulfonyl fluorides (**45**) and benzene-1,3,5-trisulfonyl fluorides (**47**) undergo nucleophilic aromatic substitution of the -SO_2_F group at high temperature (150–240 °C). The reaction proceeds via displacement of one (or up to two) of the sulfonyl fluoride groups by fluoride ion to give the corresponding aryl fluorides (**44, 46, 48, 49**) in moderate to good yields (49–80%). Although the reaction is suggested to only require catalytic amounts of fluoride ion, the authors concede that good conversions and yields almost always required the addition of at least one full equivalent of fluoride ion. This stoichiometric requirement for fluoride ion rules out this reaction, in its current form, as a possible method for synthesis of n.c.a. [^18^F] sulfonyl fluorides. If the method could be made truly catalytic in fluoride ion, unfortunately the presence of sulfonyl fluoride functional groups would still facilitate rapid ^18^F/^19^F exchange, and essentially, the method could only ever be used as a c.a. ^18^F-fluorination method. The method would need to start with a different precursor, such as a sulfonyl chloride, and rely upon an [^18^F]sulfonyl fluoride being generated as an intermediate, to be potentially applicable as a n.c.a. method.

### SF_5_ functional group

**Fig. 14:**
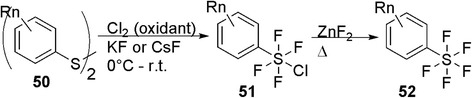
Synthesis of pentafluorosulfanyl arenes

The ever-growing interest in functional groups that can flexibly modify the pharmaceutical features of known and new drugs, has recently revived the focus on perfluorinated sulfur groups, SF_3_ and SF_5_. Especially the latter one, the pentafluorosulfanyl group, was already demonstrated half a century ago (Sheppard 1962) of its impressive stability and compatibility with several reactions. This moiety has been characterized in detail (Bowden et al. 2000), and its stability is comparable or superior to CF_3_ while being more lipophilic but also more electronegative. These typically contrasting features, shared with few other important fluorinated groups (CF_3_, OCF_3_, SCF_3_), can open up interesting avenues in the design of new drug leads. The features, synthesis and utilization of the pentafluorosulfanyl groups have recently been reviewed (Savoie and Welch 2015). Its synthesis has been improved from the original reports, and the most effective route for arene functionalization (Umemoto et al. 2012) passes from oxidative nucleophilic fluorination of arenedisulfides (**50**) or arenethiophenols, leading to the corresponding arenesulfur chlorotetrafluoride (**51**, Fig. 14). This intermediate that can be isolated, is then transformed in the target pentafluorosulfanylarene by nucleophilic fluorination in slightly harsher conditions (e.g. higher temperature, different fluoride sources).

In summary, the SF_5_ group can be prepared only via nucleophilic sources of fluorine; it is envisaged that such moiety could represent a useful ^18^F functional group which incorporates five fluorine atoms and therefore, like in the case of the trifluoroborate moiety (Liu et al. 2013), provide a way to increase the maximum molar radioactivity of ^18^F-radiopharmaceuticals up to five times. In addition, the heightened practice of introducing this functionality in new drugs will open the opportunity and the interest to access a wider range of radiopharmaceuticals labelled with an ^18^F version of the SF_5_ group.

### Use of SF_6_ as fluorinating agent

**Fig. 15:**
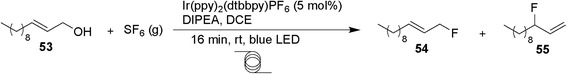
Deoxyfluorination using SF_6_ under continuous flow and photochemical conditions

Sulfur hexafluoride (SF_6_) is an inexpensive, inert gas with many industrial applications. However, it has only just recently been reported as a source of fluorine atoms for the deoxyfluorination of primary and secondary allylic alcohols (McTeague and Jamison 2016). In the presence of a photocatalyst and under blue LED light, the overnight reaction between an alcohol derivative (**53**) and SF_6_ yielded two fluorinated isomers, predominately the linear isomer (**54**), rather than the branched one (**55**), in the majority of the analogues tested in the substrate scope. The fluorination reaction was then evaluated under continuous flow conditions in an attempt to decrease the reaction time. A solution of the alcohol, photocatalyst and base (DIPEA) were pumped into a Y-mixer, where it met with a stream of SF_6_ gas from another channel. The two phases were reacted at room temperature for 1 min, before flowing through to another loop illuminated by blue LED light for 15 min. Under flow conditions, the allylic fluoride (**54**, Fig. 15) was produced in 79% yield in a total of 16 min, compared to 55% yield under batch conditions (14 h reaction time). The ratio of the linear to branched isomers also slightly increased under flow conditions (1.3:1 vs. 1.2:1 under batch conditions). These results, in particular, under continuous flow conditions, demonstrate the potential applications of SF_6_ as a fluorinating agent in organic synthesis and potentially could be more readily used in ^18^F-fluorination chemistry due to the potential availability of cyclotron produced [^18^F] SF_6_.

### Deoxyfluorination mediated by aryl fluorosulfonates

**Fig. 16:**
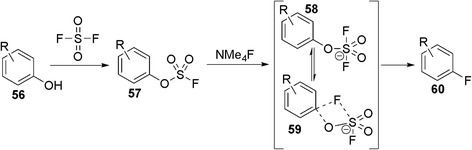
Deoxyfluorination using arylfluoro sulfonate intermediates and the proposed mechanism

Recent work by the Sanford group (Schimler et al. 2017), reported a new deoxyfluorination strategy, passing from arylfluoro sulfonates (Fig. 16).

The authors first synthesized a series of sulfonates **57** and evaluated these as substrates in nucleophilic fluorination reactions. In these experiments they found out that the *ipso* fluorination was generally favoured compared to substitution to NO_2_ or Cl nucleofuges, with overall reactivity still governed by the nature of *para-* and *ortho-* electron-withdrawing groups. A one-pot procedure was also tested, in which a substituted phenol (**56**) was successfully reacted with sulfuryl fluoride (SO_2_F_2_) and a nucleophilic source of fluoride, affording the desired fluorinated arenes (**60**) in good yield under mild conditions. The proposed mechanism, supported by calculations and product distribution analysis, involves a pentacoordinate intermediate (**58, 59**), which is formed via the attack of a fluoride anion on the sulfur centre, and its subsequent rearrangement to the fluoroarene product. It is likely that the potential of this route for translation into ^18^F chemistry will be investigated, possibly by modifying the precursor composition which will affect and allow optimisation of the steric/electronic characteristics of the proposed transition state.

## Conclusions

The use of sulfur-fluoride bonds represents a growing trend in drug development, hampered by the discovery of the substantial stability of several moieties containing such bond. It is therefore forecasted that the investigation of creating these bonds with ^18^F will be of relevance in the future of radiopharmaceutical field. Added to this, the specific reactivity of the S-F bond has facilitated the development of several fluorinating agents currently employed in traditional organic chemistry. The thorough understanding of their working principles may provide access to new mild and selective radiofluorinating agents for potential use in late-stage labelling.
